# Large B-cell Lymphoma With Interferon Regulatory Factor 4 Rearrangement Presenting as a Primary Central Nervous System Lymphoma in the Brain

**DOI:** 10.7759/cureus.83753

**Published:** 2025-05-08

**Authors:** Hannah Cutshall, Vijay M Patel, Philip E Ferguson, Rebecca A Levy, Murat Gokden

**Affiliations:** 1 Department of Pathology, University of Arkansas for Medical Sciences, Arkansas, USA; 2 Department of Pathology, Northwest Arkansas Pathology Associates, Arkansas, USA

**Keywords:** brain, diffuse large b-cell lymphoma, irf4 rearrangement, large b-cell lymphoma, primary cns lymphoma

## Abstract

Large B-cell lymphoma (LBCL) with interferon regulatory factor 4 (*IRF4*)* *rearrangement (LBCL-*IRF4*r) is a rare type of lymphoma with an overall favorable prognosis, and has been included as a distinct entity in the 2022 revision of the World Health Organization Classification of Lymphoid Neoplasms. More common in pediatric/young adult populations and rare in older adults, it typically presents in the head and neck, specifically, the Waldeyer ring, and rarely in other sites. To our knowledge, it has not been reported as a primary central nervous system (CNS) lymphoma in the English language medical literature. Although *IRF4* rearrangement can be seen in other hematolymphoid neoplasms, their histology and immunophenotype differ greatly. Familiarity with such unusual entities is important for pathologists in the work-up and diagnosis of hematolymphoid neoplasms of the CNS and is especially critical with this entity due to overall better prognosis and possible targeted treatments in the future. Here, we present a case of LBCL-*IRF4*r presenting as a primary CNS lymphoma in the brain.

## Introduction

Large B-cell lymphoma (LBCL) with interferon regulatory factor 4 (*IRF4*) rearrangement (LBCL-*IRF4*r) is a rare form of lymphoma, constituting less than 0.1% of LBCL [1]. It was previously codified as a variant of follicular lymphoma in the WHO Classification Hematolymphoid Tumors, 4th edition [2], but reclassified as LBCL due to its large cell morphology [3]. It is also listed as a definite entity in the new International Consensus Classification (ICC) of mature lymphoid neoplasms [4].

Its incidence has been reported to be up to 21% in the pediatric age group, with no definitive gender predilection [5], but it is rare in the adult population [6]. LBCL-*IRF4*r typically presents in the lymphoid structures in the Waldeyer ring, cervical lymph nodes, or bowel. Additional locations, such as skin/soft tissue or axillary/inguinal lymph nodes, have been reported in adult populations [7], but no reports of central nervous system involvement as a primary CNS lymphoma exist to our knowledge in the English language medical literature.

This article was previously presented as a meeting abstract at the 100th Annual Scientific Meeting of the American Association of Neuropathologists on June 6-9, 2024.

## Case presentation

A man in his early 30s presented with headache, double vision, and dizziness. Radiologic examination identified multiple basal ganglionic enhancing lesions measuring between 0.7 cm and 1.3 cm, with surrounding edema.

**Figure 1:**
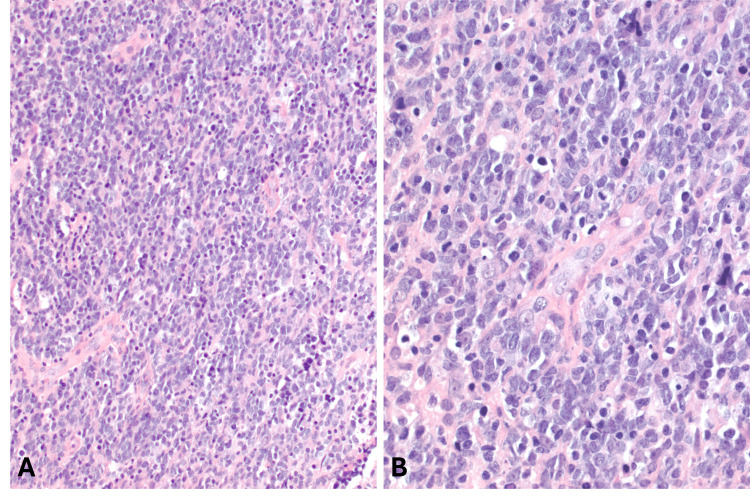
Histologic findings (A) Sheets of atypical lymphoid cells. (B) Irregular hyperchromatic nuclei of intermediate size. (Original magnifications: A, 200x; B, 400x)

Biopsy showed diffuse sheets of large lymphoid cells with mildly irregular nuclei, open/vesicular chromatin, and occasional nucleoli/chromocenters. No distinct follicular architecture was seen (Figure 1A, 1B).

**Figure 2:**
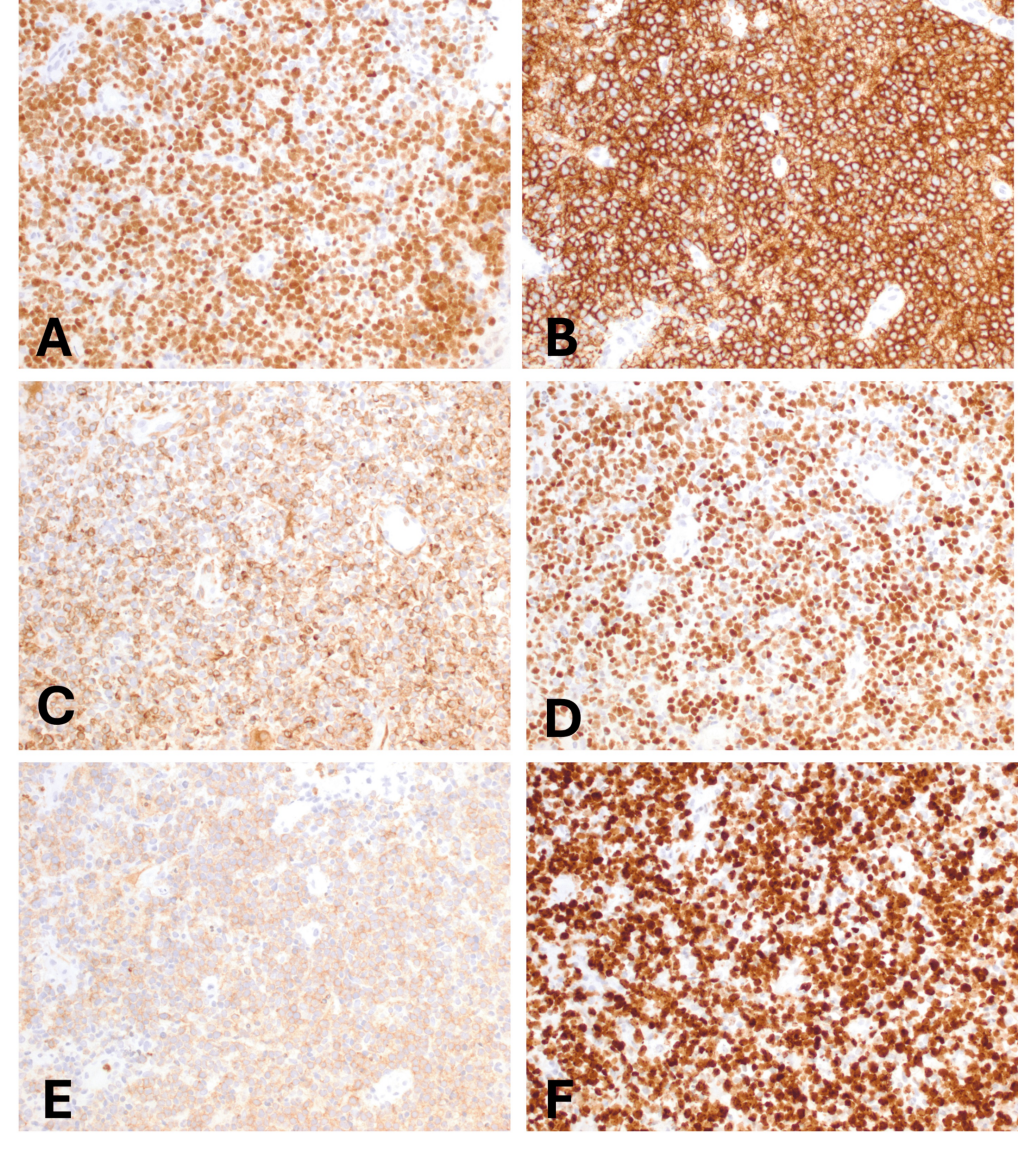
Immunohistochemical findings The neoplasm is positive for (A) MUM-1, (B) CD20, (C) BCL2, (D) BCL6, and (E) CD10, with a high Ki-67 proliferation index (F). (Original magnifications: A-F, 200x)

Immunohistochemical stains were positive for MUM1, CD20, BCL6, BCL2, and CD10, and Ki-67 proliferation index was about 90% (Figure 2A-2F). The neoplastic cells were negative for CD3, CD5, cyclin D1, CD30, and EBER in situ hybridization.

**Figure 3:**
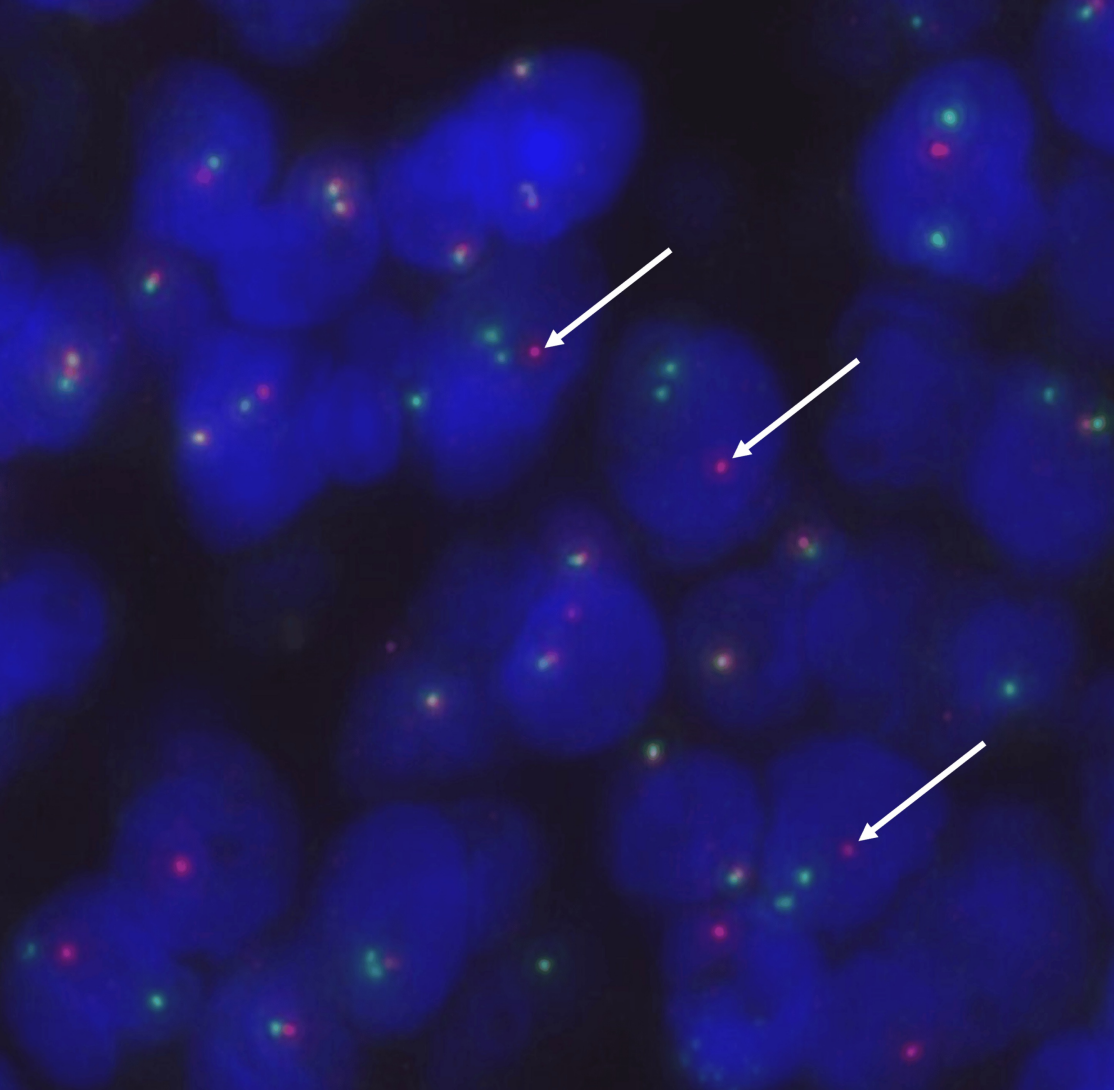
Fluorescence in situ hybridization findings Dual-color break apart probe (green: DUSP22; red: IRF4): Multiple green signals and single red signal (arrows) was identified in 84.0% nuclei (normal <11.6%).

Fluorescence in situ hybridization (FISH) identified a *DUSP22::IRF4* rearrangement by dual color dual fusion probes (Figure 3), with no rearrangements of *BCL2*, *BCL6*, or *MYC*.

Flow cytometry of the cerebrospinal fluid was negative for an abnormal T-cell population, with essentially absent B cells. Bone marrow biopsy was negative for lymphoma by morphology and flow cytometry. Positron Emission Tomography (PET) and Computed Tomography (CT) showed no evidence of systemic disease. The diagnosis of primary central nervous system (CNS) LBCL-*IRF4*r was made. The patient received four cycles of a combination chemotherapy consisting of methotrexate, cytarabine, thiotepa, and rituximab (MATRIX) with significant response, followed by BCNU-Thiotepa-based autologous stem cell transplant. No evidence of disease was found upon radiologic evaluation. He is in complete remission three years after initial diagnosis.

## Discussion

IRF4 is an interferon regulatory family transcription factor that is also known as MUM1 and is located at the 6p25.3 gene locus. It is involved primarily in B-cell maturation and differentiation, which makes it a key mutation driver in the development of plasma cell neoplasms. *IRF4* mutations also act as drivers in diffuse large B-cell lymphoma (DLBCL), specifically the activated B-cell (ABC) class through the NF-κB pathway. However, while *IRF4* point mutations can be involved in ABC class DLBCLs, the rearrangement leads to a germinal center B-cell-like DLBCL, with activation of the NF-κB pathway [6,8].

Histopathology of this entity can show either follicular or diffuse architecture. Cytologically, the cells are often intermediate-to-large with small nucleoli. In cases of follicular growth pattern, a high-grade follicular lymphoma could be considered in the differential diagnosis [9]. In addition to high grade lymphomas with diffuse and follicular patterns, *IRF4*r has also been identified in plasma cell myeloma [10], peripheral T-cell lymphoma [11], ALK-negative anaplastic large cell lymphoma (including primary cutaneous and breast implant-associated types) [12,13], and chronic lymphocytic leukemia [14]; however, the morphology and immunophenotype of these entities are significantly different, aiding in their accurate identification. DLBCLs can be further characterized using the Hans algorithm based on staining patterns of CD10, MUM1 and BCL6 as having germinal center (CD10 positive or negative, BCL6 positive, MUM1 negative) or activated B-cell (CD10 negative, BCL6 positive or negative, MUM1 positive) phenotype. Strong co-expression of IRF4/MUM1 and BCL6/CD10 is characteristic of LBCL-*IRF4*r, distinguishes it from the typical Hans algorithm, and is a clue for further investigation [15]. In addition to the morphological overlap between the DLBCL and LBCL-*IRF4*r, mutations of the NF-κB pathway-related genes, such as *MYD88* and *CD79B*, can be present in about 35% of cases [1], which also overlaps with DLBCL [16].

Several large series and case reports are available on LBCL-*IRF4*r. The largest of these reported involvement of the lung, thyroid gland, and tongue (in addition to the usual sites) [17]. One series highlighted 43 cases, including 31 adult cases, with various locations including Waldeyer’s ring, cervical lymph nodes, small bowel, and skin/soft tissue. The adult cases more frequently presented with atypical morphology and at unusual sites, while all pediatric cases presented in the usual sites [7]. Another series, which highlighted 13 cases with varying follicular, diffuse, or mixed growth patterns, additionally identified cases in the stomach, salivary gland, nasal cavity, and tongue [18]. One case of CNS ALK-negative anaplastic large cell lymphoma with *IRF4::DUSP22* rearrangement in a 55-year-old man is on record [19]. Three cases of Epstein-Barr virus (EBV)-positive DLBCL with IRF4r have recently been reported, one being in the thalamus of a 64-year-old woman who died before starting treatment [20]. It was Ann Arbor stage IV and had ABC phenotype (CD10 negative, BCL6 positive, MUM-1 positive). It is interesting to note that both of these cases in the brain, the recently-reported one in the thalamus and our case in the basal ganglia, involved deep grey matter structures; however, no such conclusion can be drawn based only on two cases. Otherwise, no reports of a bona fide LBCL-*IRF4*r presenting as a primary CNS lymphoma are identified in the English literature.

Although treatment is not significantly different from the approach to DLBCL, the overall prognosis of LBCL-IRF4r is favorable in pediatric/young adult population [1,6]; however, due to the rarity of cases, no definitive prognostic data are present for adult patients with a re-screening of 237 lymphomas previously diagnosed as DLBCL was able to identify only five cases (2%) of LBCL-*IRF4*r [17]. Awareness of this entity and a high index of suspicion should help with identification during routine diagnostic workup. Histologic differential diagnosis includes other high-grade lymphomas, especially DLBCL. As stated, paying close attention to immunophenotypic features will alert the pathologist to pursue additional testing by FISH to investigate for *IRF4*r. If identified, it should be kept in mind that this rearrangement has also been reported in the CNS rarely in other high-grade lymphomas as discussed above.

## Conclusions

LBCL-*IRF4*r is a rare type of lymphoma included as a distinct entity in the WHO Classification of Hematolymphoid Tumors and in the ICC of mature lymphoid neoplasms. Although superficially similar to DLBCL, its intermediate-size cells and immunohistochemical divergence from the Hans classification are clues to the possibility of this diagnosis, which is important due to an overall favorable prognosis (especially in the pediatric and young adult populations). Unusual sites outside the Waldeyer ring are increasingly recognized, but no reports in the CNS exist to our knowledge in the English language medical literature. Here, we report the first case of LBCL-*IRF4*r presenting as a primary CNS lymphoma, and discuss differential diagnosis and pitfalls. It is critical for pathologists to be familiar with this entity, particularly those who typically are involved in the work-up and diagnosis of hematolymphoid neoplasms involving the CNS.
